# The relationship between Ki-67 expression and imaging signs and pathological features in GISTs

**DOI:** 10.3389/fsurg.2023.1095924

**Published:** 2023-03-08

**Authors:** Lin Xiao, Yiding Zhang, Yajie Wang, Lede Liu, Yisheng Pan

**Affiliations:** Department of General Surgery, Peking University First Hospital, Beijing, China

**Keywords:** gastrointestinal stromal tumor, Ki-67 expression index, plain-scan CT signs, pathological features, nomogram

## Abstract

**Introduction:**

To investigate the correlations between the Ki-67 index and plain-scan computerized tomography (CT) signs and pathological features of gastrointestinal stromal tumor (GIST) tissue.

**Materials and methods:**

Data from 186 patients with GIST diagnosed by pathology and immunohistochemistry (IHC) in Peking University First Hospital from May 2016 to May 2022 were analyzed. The patients were divided into two groups: Ki-67 ≤5% and >5%. Correlation analysis, univariate and multivariate Logistic regression analysis were used to explore the correlations between CT signs, pathological features, and Ki-67 expression.

**Results:**

Univariate indicators correlated with the Ki-67 index were mitotic count, pathological grade, tumor hemorrhage, tumor necrosis, tumor size, and tumor density. Multivariate Logistic regression indicated that the mitotic count [odds ratio (OR) 10.222, 95% confidence interval (CI) 4.312–31.039], pathological grade (OR 2.139, 95% CI 1.397–3.350), and tumor size (OR 1.096, 95% CI 1.020–1.190) were independently associated with the Ki-67 expression level. The concordance indexes (C-index) for the pathological features and CT signs models were 0.876 (95% CI 0.822–0.929) and 0.697 (95% CI 0.620–0.774), respectively, with positive predictive values of 93.62% and 58.11% and negative predictive values of 81.29% and 75.89%, respectively. After internal verification by the Bootstrap method, the fitting degree of the pathological features model was found to be better than that of the CT signs model.

**Conclusion:**

Mitotic count, pathological risk grading, and tumor size are independent risk factors correlating with high Ki-67 index. These results indicate that the Ki-67 index reflects tumor malignancy and can predict recurrence and metastasis of GIST.

## 1. Introduction

Gastrointestinal stromal tumor (GIST) was first discovered by Mazur and Clark in 1983. GIST accounts for approximately 1%–2% of all gastrointestinal tumors (1). GIST can occur in any part of the gastrointestinal tract, with the stomach being the most common location, accounting for 60% of all GISTs. The small intestine is the second most common GIST site, accounting for 20%–30% of all GISTs (2). While the incidence of GIST is relatively low, the many non-specific and atypical clinical symptoms of GIST together with the complex and diverse imaging manifestations, including intestinal thickening and overlapping intestinal loops, make it extremely difficult to accurately diagnose GIST. Thus, delayed or misdiagnosis of GIST commonly occurs (3). Treatment of GISTs includes surgery and medication, and the standard treatment for localized GISTs is complete surgical resection, known as R0 resection. The use of tyrosine kinase inhibitors (TKIs) has revolutionized the treatment of GISTs because imatinib has been effective in inhibiting tumor growth as a first-line therapy (4, 5).

Ki-67 is a protein phosphatase 1 (PP1) binding protein that organizes the mitotic chromosome; it plays a role in the phosphorylation regulation of nucleolar protein B23/nucleophosmin. Ki-67 is rarely expressed in normal cells but often expressed in malignant cells; thus, it is widely used as an important indicator for evaluating the proliferation status of tumor cells and judging tumor prognosis (6). Some studies have found that Ki-67 is an important prognostic factor for the recurrence of GIST, indicating that the Ki-67 index is of great significance for evaluating the malignancy potential of the disease (7). To date, many studies have evaluated the risk of recurrence and metastasis of GIST using the Ki-67 index. However, few clinical studies have investigated the correlations between the Ki-67 index and computed tomography (CT) signs and pathological features of GIST. In this study, the associations between Ki-67 expression in GIST tissue and (1) the non-enhanced CT imaging manifestations of GIST and (2) the pathological features of the disease were examined *via* correlation analysis.

## 2. Materials and methods

### 2.1. Population

#### 2.1.1. Research population

Between May 2016 and May 2022, 186 patients with suspected GIST underwent preliminarily examination with a 128-slice CT machine (GE discovery 750) followed by GIST diagnosis confirmation by pathology and immunohistochemistry (IHC) examinations at Peking University First Hospital.

#### 2.1.2. Inclusion criteria

1) Neoplasm diagnosed as GIST by postoperative pathology.
2) IHC examination and tumor risk assessment of the neoplasm.
3) Tumor located in the duodenum, stomach, jejunum, or ileum.
4) Complete imaging data available.
5) Complete pathology data available.
6) Primary GIST and no other treatments received before surgery.

#### 2.1.3. Exclusion criteria

1) Incomplete pathological, IHC, or imaging data.
2) Antitumor therapy received before surgery and CT examination.

#### 2.1.4. Ethics

The protocol for this study was approved by the Human Ethics Committee of Peking University First Hospital. All patients were given a verbal and written explanation of the study and written informed consent was obtained. No personal information was recorded during the research. This study was conducted in accordance with the Declaration of Helsinki.

### 2.2. Research materials

#### 2.2.1. General data

Gender and age of the patients.

#### 2.2.2. Imaging data

1) Tumor location: duodenum, stomach, jejunum, or ileum.
2) Tumor size: the longest diameter of the tumor.
3) Tumor morphology: lobulated or round.
4) Tumor growth pattern: intraluminal or luminal.
5) Whether the surrounding tissue was infiltrating or metastatic.
6) Whether the density of the plain CT scan was uniform.
7) Whether the tumor boundary was clear.

#### 2.2.3. Pathology data

1) Mitotic count.
2) Pathological risk grading.
3) Tumor tissue necrosis.
4) Tumor tissue hemorrhage.

The patients were divided into two groups based on their Ki-67 index: Ki-67 index ≤5% (group A) and >5% (group B).

### 2.3. Statistical analysis

The Shapiro-Wilk test was used to test the normality of the data. The data that were normally distributed were described using the mean ± standard deviation (SD), while non-normally distributed continuous data were described using the median and interquartile range (IQR). *T*-tests were used to evaluate the differences in the normally distributed measurement data between the two groups; non-normally distributed continuous data were evaluated by the Wilcoxon rank sum test. The *χ*^2^ test or Fisher's exact test was used to compare the differences in the categorical variables between the groups. A *P*-value <0.05 (two-sided) was considered statistically significant.

To analyze the independent predictors of the Ki-67 index, binary logistic regression was conducted. Univariable logistic regression was used to preliminarily evaluate the independent predictors of the Ki-67 index, and then multivariable logistic regression analysis was conducted with the inclusion of the statistically significant univariate predictors.

The predictive performance of the logistic regression models was verified by the likelihood ratio test combined with the Hosmer and Lemeshow test. The optimal cutoff value was calculated by drawing a receiver operating characteristic (ROC) curve. The area under the curve (AUC), accuracy, sensitivity, specificity, positive predictive value, and negative predictive value of the prediction models were also evaluated.

Based on the results of the multivariable logistic regression analyses, the rms package (version 6.3) in R software was used to draw nomograms. In the nomograms, each coefficient of the multivariate logistic regression was proportionally converted to a score between 0 and 100, and the variable with the highest beta coefficient was set to a score of 100. The scores corresponding to the independent factors were added together to obtain a total score, and each total score corresponded to a probability value, which was the predicted probability value obtained by the prediction model. The corresponding predicted probability value can only be obtained by calculating the total score through the nomogram in clinical practice. In this study, the predictive power of each nomogram was measured by calculating the C-index and conducting internal validation with the Bootstrap method.

All statistical analysis was conducted in R software (version 4.2.1).

## 3. Results

### 3.1. Clinical data

A total of 186 patients were enrolled in this study, including 100 males (53.8%) and 86 females (46.2%) (male:female ratio of 1.16:1). The patients ranged in age from 34 to 85 years, with an average age of 62.04 ± 10.48 years. The primary tumor was located in the stomach in 131 patients (70.4%), jejunum in 25 patients (13.4%), ileum in 18 patients (9.7%), and duodenum in the remaining 12 patients (6.5%). According to the 2008 modified National Institutes of Health (NIH) risk classification criteria, 20 patients (10.8%) had a very low risk, 73 patients (39.2%) had a low risk, 36 patients (19.4%) had an intermediate risk, and 57 patients (30.6%) had a high risk. The clinical data for the enrolled patients are shown in Table 1.

**Table 1:** Clinical data of all patients.

Variable		Level	Overall (186 cases)
General characteristics	Age [median (IQR)]	63.00 [54.00, 70.75]	
	Gender (%)	Female	86 (46.2)
		Male	100 (53.8)
Imaging characteristics	Tumor imaging size [median (IQR)]	4.35 [3.00, 7.35]	
	Location (%)	Jejunum	25 (13.4)
		Ileum	18 (9.7)
		Stomach	131 (70.4)
		Duodenum	12 (6.5)
	Growth mode (%)	Intracavity	57 (30.6)
		Extra cavity	92 (49.5)
		Mixed	37 (19.9)
	Metastasis (%)	N	168 (90.3)
		Y	18 (9.7)
	Morphology (%)	Lobular	106 (57.0)
		Round	80 (43.0)
	Density (%)	N	88 (47.3)
		Y	98 (52.7)
	Boundary (%)	N	31 (16.7)
		Y	155 (83.3)
Pathological characteristics	Blooding (%)	N	111 (59.7)
		Y	75 (40.3)
	Necrosis (%)	N	124 (66.7)
		Y	62 (33.3)
	Mitotic count (%)	≤5	139 (74.7)
		6–10	22 (11.8)
		>10	25 (13.4)
	Pathological risk grading (%)	Very low	20 (10.8)
		Low	73 (39.2)
		Moderate	36 (19.4)
		High	57 (30.6)

IQR, interquartile range.

### 3.2. Comparison of the Ki-67 subgroups

A comparison of the CT signs and pathological features between the two groups is shown in Table 2. The results showed that the tumor size of group B was larger than that of group A [6.20 (4.12, 9.18) vs. 3.80 (2.38, 5.62), *P* < 0.001]. The most prevalent mitotic count was ≤5/50HPF (high power field) in both groups (97.4% vs. 37.1%), but the proportions of 6–10/50HPF and >10/50HPF were significantly higher in group B (30.0% vs. 0.9%, 32.9% vs. 1.7%, *P *< 0.001). The patients in group B were more likely to have a heterogeneous tumor density (61.4% vs. 38.8%, *P* = 0.003), tumor hemorrhage (55.7% vs. 31.0%, *P* = 0.001), necrosis (48.6% vs. 24.1%, *P* = 0.001), and other poor pathological performance indicators. Further, group A had a higher proportion of low-risk patients (54.3% vs. 14.3%), while group B had a higher proportion of high-risk patients (54.3% vs. 16.4%) (*P* < 0.001).

**Table 2:** Comparison of the Ki-67 subgroups.

Variable		Level	≤5% (116 cases)	>5% (70 cases)	*P*
General characteristics	Age [median (IQR)]		63.00 [54.00, 71.25]	62.00 [55.00, 68.75]	0.637
	Gender (%)	Female	53 (45.7)	33 (47.1)	0.847
		Male	63 (54.3)	37 (52.9)	
Imaging characteristics	Image size [median (IQR)]		3.80 [2.38, 5.62]	6.20 [4.12, 9.18]	**<0** **.** **001**
	Location (%)	Jejunum	17 (14.7)	8 (11.4)	0.305
		Ileum	8 (6.9)	10 (14.3)	
		Stomach	82 (70.7)	49 (70.0)	
		Duodenum	9 (7.8)	3 (4.3)	
	Growth (%)	Intracavity	40 (34.5)	17 (24.3)	0.107
		Extra cavity	58 (50.0)	34 (48.6)	
		Mixed	18 (15.5)	19 (27.1)	
	Metastasis (%)	N	107 (92.2)	61 (87.1)	0.255
		Y	9 (7.8)	9 (12.9)	
	Morphology (%)	Lobular	60 (51.7)	46 (65.7)	0.062
		Round	56 (48.3)	24 (34.3)	
	Density (%)	N	45 (38.8)	43 (61.4)	**0** **.** **003**
		Y	71 (61.2)	27 (38.6)	
	Boundary (%)	N	15 (12.9)	16 (22.9)	0.078
		Y	101 (87.1)	54 (77.1)	
Pathological characteristics	Blooding (%)	N	80 (69.0)	31 (44.3)	**0** **.** **001**
		Y	36 (31.0)	39 (55.7)	
	Necrosis (%)	N	88 (75.9)	36 (51.4)	**0** **.** **001**
		Y	28 (24.1)	34 (48.6)	
	Mitotic count (%)	≤5	113 (97.4)	26 (37.1)	**<0** **.** **001**
		6–10	1 (0.9)	21 (30.0)	
		>10	2 (1.7)	23 (32.9)	
	Pathological risk grading (%)	Very low	19 (16.4)	1 (1.4)	**<0** **.** **001**
		Low	63 (54.3)	10 (14.3)	
		Moderate	15 (12.9)	21 (30.0)	
		High	19 (16.4)	38 (54.3)	

IQR, interquartile range; bold values: *P* < 0.05.

### 3.3. Logistic regression analysis of the expression of Ki-67 in patients with GIST

Univariate logistic regression analyses were carried out with the above variables. Mitotic count (OR 16.657, 95% CI 7.164–49.916, *P* < 0.001), pathological risk grading (OR 3.485, 95% CI 2.416–5.206, *P* < 0.001), tumor hemorrhage (OR 2.796, 95% CI 1.521–5.208, *P* = 0.001), and necrosis (OR 2.968, 95% CI 1.584–5.635, *P* = 0.001) were pathological features associated with the expression level of Ki-67 in patients with GIST. Imaging tumor size (OR 1.128, 95% CI 1.054–1.217, *P* = 0.001) and tumor density (OR 0.398, 95% CI 0.214–0.727, *P* = 0.003) were the CT signs that were associated with the expression level of Ki-67 in patients with GIST (Table 3). The above variables were then incorporated into multivariate logistic regression analyses and were screened by stepwise regression. Specifically, there were separate regression models for CT signs and pathological features (Table 4). The mitotic count (OR 10.222, 95% CI 4.312–31.039, *P* < 0.001), pathological risk grading (OR 2.139, 95% CI 1.397–3.350, *P* = 0.001), and tumor imaging size (OR 1.096, 95% CI 1.020–1.190, *P* = 0.018) were independently associated with the Ki-67 expression level in our prediction models.

**Table 3:** Results of univariate logistic regression analysis.

Variable		*β*	OR (95% CI)	*P*
Age		−0.001	0.999 (0.971–1.028)	0.958
Gender = Male		−0.058	0.943 (0.520–1.713)	0.847
Location	Jejunum = Y	−0.286	0.751 (0.291–1.797)	0.533
	Ileum = Y	0.811	2.250 (0.844–6.187)	0.105
	Stomach = Y	−0.033	0.967 (0.508–1.868)	0.921
	Duodenum = Y	−0.631	0.532 (0.115–1.857)	0.357
Tumor imaging size		0.120	1.128 (1.054–1.217)	**0** **.** **001**
Growth mode	Intracavity = Y	−0.495	0.609 (0.307–1.174)	0.146
	Extra cavity = Y	−0.057	0.944 (0.521–1.711)	0.850
	Mixed = Y	0.707	2.028 (0.978–4.229)	0.057
Metastasis = Y		0.562	1.754 (0.652–4.724)	0.259
Morphology	Lobular = Y	0.582	1.789 (0.975–3.336)	0.063
	Round = Y	−0.582	0.559 (0.300–1.026)	0.063
Density = Y		−0.921	0.398 (0.214–0.727)	**0** **.** **003**
Boundary = Y		−0.691	0.501 (0.228–1.094)	0.082
Blooding = Y		1.028	2.796 (1.521–5.208)	**0** **.** **001**
Necrosis = Y		1.088	2.968 (1.584–5.635)	**0** **.** **001**
Mitotic count		2.813	16.657 (7.164–49.916)	**<0** **.** **001**
Pathological risk grading		1.248	3.485 (2.416–5.206)	**<0** **.** **001**

OR, odds ratio; CI, confidence interval; bold values: *P* < 0.05.

**Table 4:** Results of multivariate logistic regression analysis.

Variable		*β*	OR (95% CI)	*P*
Pathological characteristics	Mitotic count	2.325	10.222 (4.312–31.039)	**<0** **.** **001**
	Pathological risk grading	0.760	2.139 (1.397–3.350)	**0** **.** **001**
Imaging characteristics	Tumor imaging size	0.092	1.096 (1.020–1.190)	**0** **.** **018**
	Density = Y	−0.555	0.574 (0.290–1.140)	0.111

OR, odds ratio; CI, confidence interval; bold values: *P* < 0.05

### 3.4. Evaluation of the prediction models

#### 3.4.1. Multivariate logistic regression of pathological features and Ki-67 index

The *P* value of the likelihood ratio test of this prediction model was less than 0.001, indicating that the OR value of at least one variable included in this model was statistically significant; that is, the overall model was statistically significant. The *P* value of the Hosmer and Lemeshow test was >0.05, indicating that this model had a good fit.

**Figure 1:**
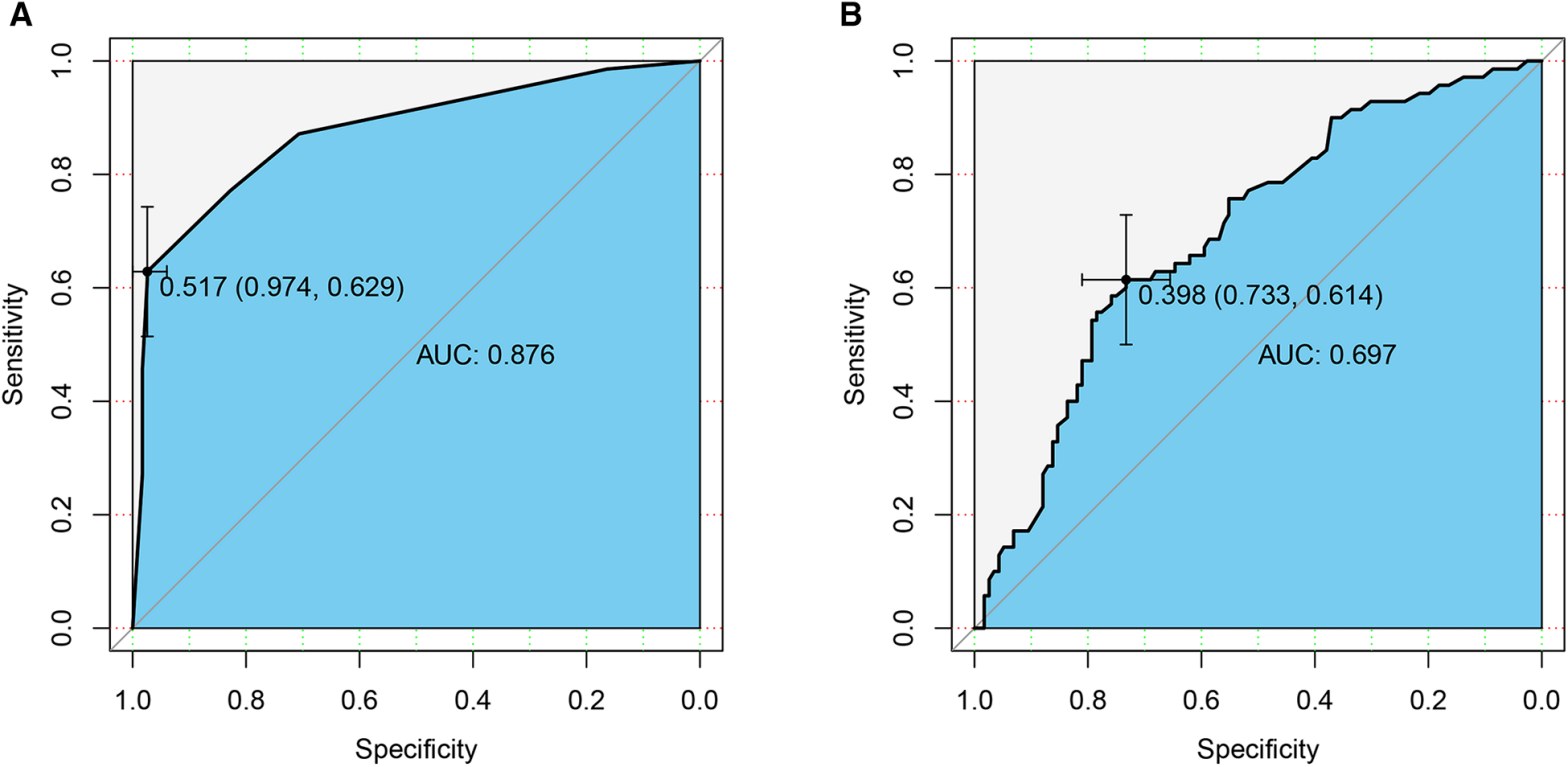
ROC curves of the prediction models. (**A**) Pathological features; (**B**) CT signs. AUC, area under the curve.

After obtaining the predicted probability, the ROC curve was drawn (Figure 1A). The AUC value was 0.876 (95% CI 0.822–0.929) and the optimal cutoff value was 0.517. According to this optimal cutoff value, prediction classification was performed. The accuracy of the model for correct classification of the dependent variable was 84.41%; the sensitivity was 62.86%, the specificity was 97.41%, the positive predictive value was 93.62%, and the negative predictive value was about 81.29%. The prediction effect of this model was good.

#### 3.4.2. Multivariate logistic regression of CT signs and Ki-67 index

The *P* value of the likelihood ratio test of the prediction model was less than 0.001 and the *P* value of the Hosmer and Lemeshow test was >0.05.

After calculating the prediction probability of this model, the ROC curve of the prediction model was drawn (Figure 1B). The AUC value was 0.697 (95% CI 0.620–0.774) and the best cutoff value was 0.398. According to this optimal cutoff value, prediction classification was performed. The accuracy of the model for the correct classification of the dependent variable was 68.82%; the sensitivity was 61.43%, the specificity was 73.28%, the positive predictive value was 58.11%, and the negative predictive value was 75.89%. The predictive effect of this model was found to be not as good as that of the pathological features model.

### 3.5. Plotting and validation of nomograms

**Figure 2:**
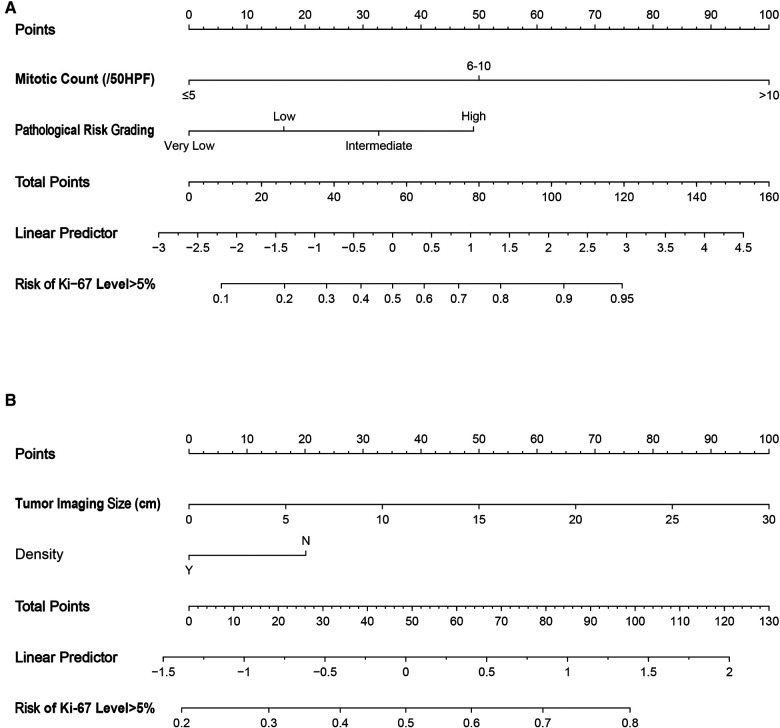
Nomograms to evaluate the risk of Ki-67 level >5% in GIST patients. Method of use: find the position of each variable on the corresponding axis, draw a line to the points axis for the number of points, add the points from all of the variables, and draw a line from the total points axis to determine the risk of Ki-67 level >5% at the lower line of the nomogram. (**A**) Pathological features; (**B**) CT signs. HPF, high power field.

Nomograms (Figure 2) were then drawn based on the above two prediction models, and internal verification was performed with the Bootstrap method.

#### 3.5.1. Validation of the pathological features models

**Figure 3:**
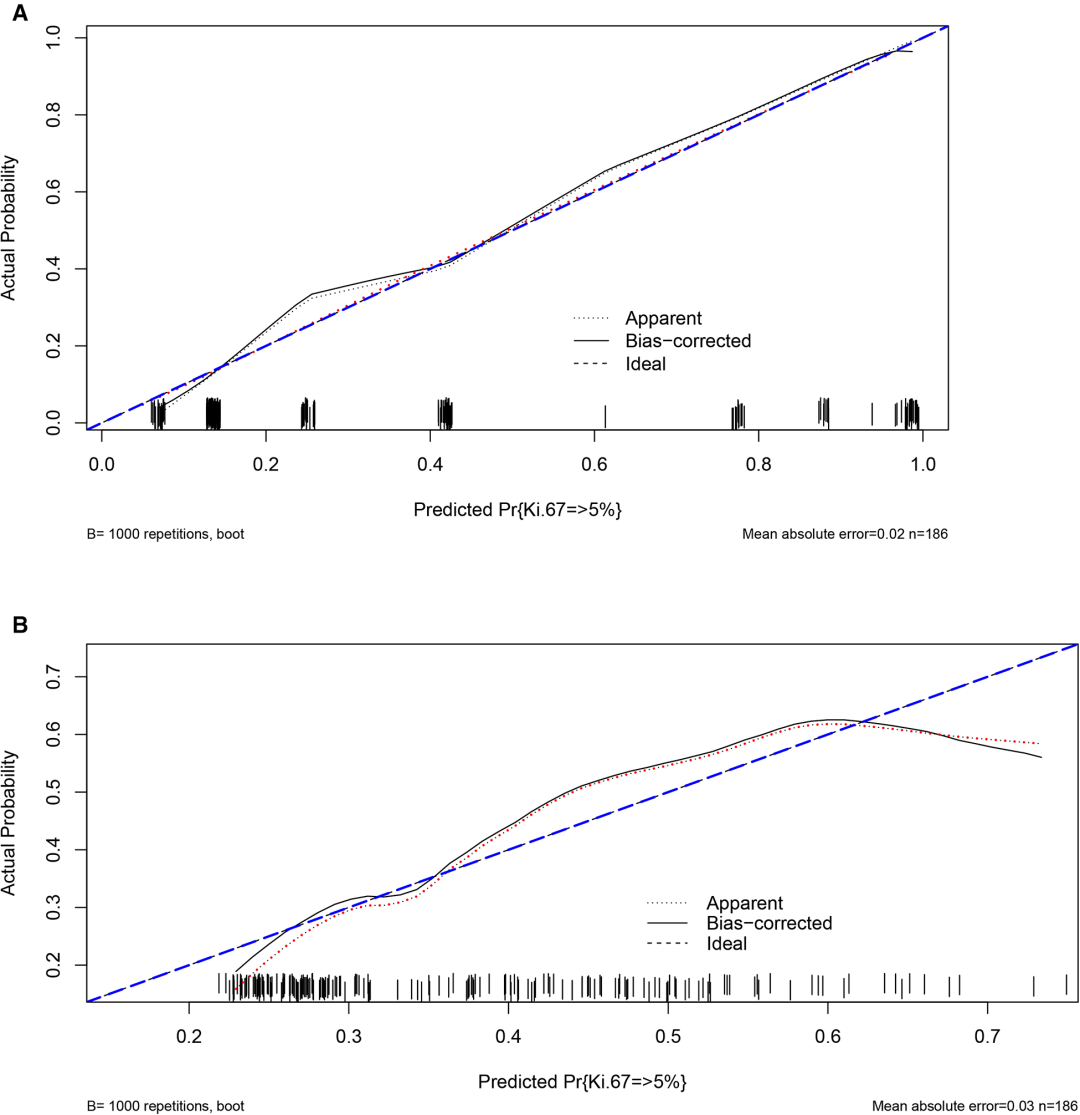
Validity of the predictive performance of the nomograms in estimating the risk of Ki-67 level >5% (*n* = 186). (**A**) Pathological features; (**B**) CT signs.

This nomogram showed good accuracy in predicting the risk of Ki-67 >5%, with an unadjusted C-index of 0.876 (95% CI 0.822–0.929) and a Bootstrap-corrected C-index of 0.876. Based on the calibration curve, it can be concluded that the predicted results calculated by this predictive model were of good value (Figure 3A).

#### 3.5.2. Validation of the CT signs models

The nomogram was generally accurate in predicting the risk of Ki-67 >5%, with an unadjusted C-index of 0.697 (95% CI 0.620–0.774) and a Bootstrap-corrected C-index of 0.689. It can be seen from the calibration curve that the results predicted by this prediction model were of average value (Figure 3B).

## 4. Discussion

GIST is the most common mesenchymal tumor of the digestive tract, accounting for 70% of all gastrointestinal mesenchymal tumors (8). It is widely believed that GIST originates from Cajal cells or their precursor cells in the gastrointestinal tract, such as mesenchymal stem cells. Research indicates that the possible pathogenesis of GIST involves abnormal activation of tyrosine kinase caused by mutation of the C-KIT gene or platelet-derived growth factor receptor alpha (PDGFRA) (9). These two mutations are mutually exclusive and are key molecular drivers of GIST proliferation, resulting in uncontrolled cell proliferation, inhibition of apoptosis, and ultimately, tumorigenesis (10, 11). GIST can occur anywhere in the digestive tract: the most common site is the stomach (50%–70%), followed by the small intestine (30%–45%); it can also be found in the colon, esophagus, and peritoneal cavity (12). GIST is usually observed in patients over 50 years of age; the mean age at final diagnosis is around 55–63 years (13). In addition, GIST has the capacity for multi-directional differentiation and has a certain malignancy potential (14). GIST is not sensitive to radiotherapy or chemotherapy. However, around 40%–65% of patients with resectable GIST experience recurrence or metastasis even after radical resection (15). With the development of tyrosine kinase inhibitors (TKIs), the treatment of GIST has improved. Sunitinib and regorafenib are approved as second- and third-line treatments for patients with GIST who develop resistance to imatinib (16, 17). However, about 5%–10% of GIST have platelet-derived growth factor receptor (PDGFRA) mutations, which lead to resistance to imatinib and sunitinib (18). Some GIST patients lack KIT and PDGFRA mutations, and approximately 20%–40% of GISTs deficient in KIT and PDGFRA mutations show succinate dehydrogenase (SDH) complex defects, and the therapeutic role of TKIs in patients with SDH-deficient GIST remains controversial. However, the study of the expression profile of GIST lacking SDH provides a new direction for the treatment strategy of GISTs (16, 19). Interestingly, in addition to the critical role that gene mutations play in GISTs, Dimino et al. also emphasized the association between tumor microenvironment (TME) and GISTs (20). The TME is mainly composed of tumor-associated macrophages and lymphocytes, and stromal differentiation has a significant impact on the prognosis and treatment response of stromal tumors. Although the influence of immune response is still unclear in GISTs, studies have identified that the expression level of PD-1 and PD-L1 is high in most GIST samples, which means the immune checkpoints and their relationship with the clinical phenotype of GISTs are becoming potential prognostic biomarkers. Looking forward, the research of TME might lead to the potential use of immunotherapy, alone or in combination with targeted therapy, in advanced refractory GISTs (21).

According to the modified NIH classification, GIST can be classified into four risk gradings—very low risk, low risk, intermediate risk, and high risk—based on tumor location (gastric or non-gastric), tumor size, and mitotic count. Mitotic count is one of the most important factors for evaluating GIST. However, since the mitotic count only reflects the M phase of cell mitosis, it cannot correctly judge the cell proliferation status (22). Ki-67 is a nuclear protein that exists only in the nucleus of proliferating cells; it can be expressed in the G1, S, G2, and M phases of cell proliferation, but not in the G0 phase (23). The Ki-67 expression index is highly related to cell proliferation and growth and has become one of the most widely used biomarkers for evaluating cell proliferation (24). Currently, the Ki-67 index is widely used to predict the proliferative potential of malignant tumors, and its potential as a reliable marker of malignant tumors has been shown in breast, lung, prostate, cervical, and central nervous system cancers (25). Studies have also shown that the expression of Ki-67 is closely related to mitotic count (23, 26, 27). The results of the current study indicated that, although the most common mitotic count in both subgroups was ≤5/50HPF, in the Ki-67 >5% group, the proportions of 6–10/50HPF and >10/50HPF patients were significantly increased. Moreover, the final multivariate logistic regression model indicated that mitotic count was an independent predictor of the expression level of Ki-67 in GIST patients. Nakamura et al. (28) argued that the Ki-67 index is a more comprehensive indicator of the degree of cell proliferation than the mitotic count.

The expression of Ki-67 is also obviously correlated with the pathological risk grading and medium- and long-term prognosis of patients with GISTs. In a clinicopathological and IHC study of GIST, it was discovered that the higher the expression level of Ki-67, the poorer the long-term prognosis of the patient and the reliability of Ki-67 for prognosis evaluation was better than other protein markers, especially in the high-risk group patients (23). In the current study, the proportions of low- and intermediate-risk patients were relatively high among the Ki-67 ≤5% group and the proportion of high-risk patients was relatively high among the Ki-67 >5% subgroup. Moreover, the multivariable logistic regression analysis indicated that pathological risk grading was an independent predictor of the expression of Ki-67. All of the aforementioned studies demonstrated that with an increase in the degree of malignancy of GIST, the proliferation of the tumor cell is more active, the growth rate of the neoplasm is faster, the invasiveness of the tumor is stronger, and the likelihood of metastasis and recurrence is greater. The Ki-67 index can serve as an important reference for evaluating the tumor risk classification. A close relationship between the expression level of Ki-67 and the prognosis of GIST was also identified in a large-sample meta-analysis, with a higher rate of Ki-67 related to worse patient prognosis (29); this is consistent with our main findings. Another large-sample retrospective analysis found that the prognosis of high-risk patients with high Ki-67 expression was significantly worse than that of patients with low Ki-67 expression (30).

CT examination is considered to be the first choice for preoperative tumor staging, surgical planning, and postoperative follow-up among GIST patients (31). In addition, existing studies suggest that the degree of malignancy of neoplasms can be further evaluated by analyzing the relationship between CT imaging features and risk stratification to optimize the preoperative therapeutic schedule and guide the surgical treatment (32). Zhou et al. (33) conducted a multivariate logistic regression analysis of CT signs in 129 GIST patients and found that tumor diameter >10 cm and mixed growth mode were independent predictors of high-risk GIST. In the current study, the tumor size of patients with higher Ki-67 expression levels was larger than that of the lower Ki-67 expression group, and the tumor size was an independent risk predictor of Ki-67 expression level among GIST patients in the multivariate logistic regression analysis. However, in the subsequent evaluation of the prediction efficiency of the model, the prediction power was not found to be high; that is, the prediction efficiency of this model still requires the support of more large-scale, multi-center studies. Nonetheless, it is undeniable that the size of the tumor on imaging is indeed related to the expression level of Ki-67. A positive correlation between Ki-67 expression and tumor size was also shown in an endoscopic ultrasonography study of GIST (34). Similarly, Li et al. (35) found that tumor size was an independent risk factor for differentiating Ki67 ≤5% and >5%, which is consistent with our findings.

Although the expression of Ki-67 in GIST patients was indeed related to patient prognosis in the current study, there is still no consensus as to the standard to distinguish the expression level of Ki-67. Belev et al. (7) performed IHC experiments and found that a Ki-67 index cutoff value of 6% was statistically significant with respect to recurrence. The authors concluded that the Ki-67 index is an important indicator for assessing prognosis and the malignant potential of the disease (7). However, Zhao et al. (36) showed that the ROC curve had high sensitivity and specificity when the Ki-67 index cut-off was 5%. In that study, a Ki-67 index >5% suggested a high risk of recurrence and poor disease-free survival (DFS) among GIST patients. The results also indicated that the Ki-67 index was an independent predictor of recurrence-free survival (RFS) among GIST patients, and that Ki-67 index >8% can complement the modified NIH criteria to distinguish between different outcomes in patients with high-risk GIST and adverse effects of adjuvant imatinib therapy. Liang et al. (37) found that GIST patients with a Ki-67 index <5% had higher DFS, while Turkel-Kucukmetin et al. (38) found that a high Ki-67 index (≥10%) was an independent predictor of poor overall survival and DFS. In summary, it is clear that the expression level of Ki-67 varies greatly among different studies, and this may be due to different authors using different cut-off values. In conclusion, Ki-67 overexpression is significantly correlated with the degree of malignancy of GIST and is an important biomarker for evaluating possible poor prognosis in GIST patients.

There are several limitations of this study that should be considered. First, since this study was a retrospective single-center study, the sample size was relatively small, and it was impossible to divide these patients into a training set and a validation set in order to conduct external validation of the prediction models. Therefore, follow-up data from further large-scale, multi-center studies are still required. Second, the predictive performance of the CT signs model for Ki-67 expression was relatively poor. In order to further clarify the predictive power of CT signs for Ki-67 expression, more studies are required. Finally, due to the lack of long-term follow-up data, we were unable to investigate the mortality of these patients.

## 5. Conclusion

This study found that mitotic count, pathological risk grading, and tumor size were independent predictors of the expression level of Ki-67. This indicates that the Ki-67 index can serve as an indicator of the degree of malignancy and can be used to predict the probability of metastasis and recurrence in GIST. It is expected that the Ki-67 index will be used as an important supplementary measure for judging GIST risk grading and evaluating prognosis in the future.

## Data Availability

The original contributions presented in the study are included in the article/Supplementary Material, further inquiries can be directed to the corresponding author.
